# Fructose: the sweet(er) side of the Warburg effect

**DOI:** 10.1038/s41418-024-01395-2

**Published:** 2024-10-05

**Authors:** Christian Frezza

**Affiliations:** 1 Cluster of Excellence Cellular Stress Responses in Aging-associated Diseases (CECAD), University Hospital Cologne, Cologne, Germany; 2 Institute of Genetics, Faculty of Mathematics and Natural Sciences, University of Cologne, Cologne, Germany

**Keywords:** Metabolomics, Cancer metabolism

Metabolic reprogramming is a hallmark of cancer, with tumours often exhibiting altered nutrient use to fuel their unchecked growth and survival. For decades, cancer metabolism research has primarily focused on glucose, especially in the context of the well-known Warburg effect, where cancer cells preferentially convert glucose into lactate even in the presence of oxygen [1]. However, recent studies have shifted attention to another sugar—fructose—showing that cancer cells can exploit fructose metabolism for tumour initiation and progression. This emerging area of research has important implications for understanding cancer biology and potentially developing novel therapeutic strategies. In a pair of back-to-back papers published in this issue of Cell Death and Differentiation, Wang et al. [2] and Zhao et al. [3] investigate the significance of fructose metabolism in cancer and propose novel strategies for its targeted intervention.

**Fig. 1:**
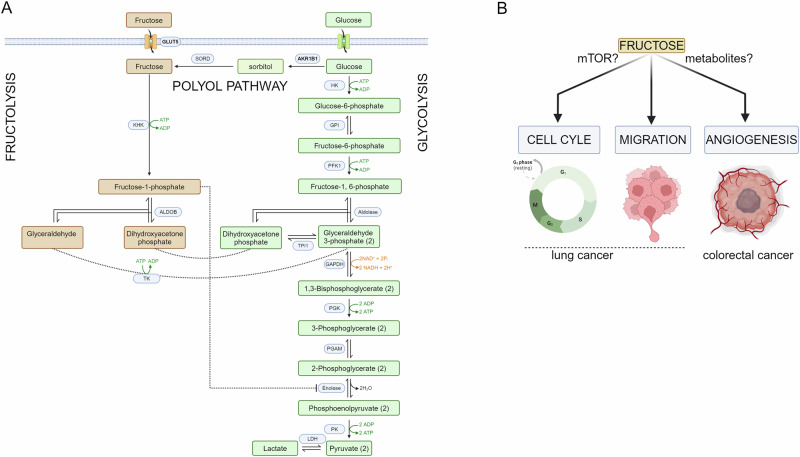
Fructose metabolism and cancer. **A** Fructose metabolism and glucose metabolism are deeply intertwined. On the one hand, fructose catabolism eventually converges towards glycolytic intermediates, namely DHAP and glyceraldehyde phosphate (GAP), converted from glyceraldehyde to GAP by TK. On the other, glucose can generate fructose via the polyol pathway. **B** In these two papers in CDD, the authors provide new connections between fructose metabolism and cell cycle, cell migration, and angiogenesis in two different tumour types. AKR1B1 aldoketoreductase, SORD sorbitol dehydrogenase, GLUT5 fructose transporter, KHK ketohexokinase; ALDOB aldolase B, TK triose kinase; HK hexokinase, GPI glucose phosphate isomerase; PFK1 phosphofructokinase; GAPH glyceraldehyde phosphate dehydrogenase, TPI1 triose phosphate isomerase, PGK phosphoglycerate kinase, PGAM phosphoglycerate mutase, PK pyruvate kinase, LDH lactate dehydrogenase.

Fructose, commonly found in fruits, honey, and processed foods (particularly in high-fructose corn syrup-containing beverages), is typically metabolised in the liver through a process distinct from glucose metabolism. Unlike glucose, fructose metabolism is not tightly regulated by insulin and follows a unique pathway called *fructolysis*. In this pathway, fructose is taken up by the dedicated transporter GLUT5, and rapidly phosphorylated by fructokinase to form fructose-1-phosphate, which is then broken down by aldolase B into intermediates shared with glucose catabolism, eventually feeding into lipid synthesis and energy production. In addition to being taken from the diet, fructose can be generated endogenously from glucose via the *polyol pathway*. The polyol pathway begins with the enzyme aldose reductase, encoded by the gene AKR1B1, which reduces glucose to sorbitol. Sorbitol is then converted to fructose by the enzyme sorbitol dehydrogenase. The resulting fructose can be further metabolised in various cellular processes. Under normal physiological conditions, the polyol pathway plays a minor role in metabolism, but in high-glucose environments—such as those seen in diabetes or cancer—it becomes more active. This dual supply of sugars via direct fructose uptake and polyol pathway activation (See Fig. 1A) could explain why certain cancers are highly aggressive and resistant to conventional therapies that target glucose metabolism alone [4]

While glucose is metabolised widely throughout the body, fructose metabolism is highly concentrated in the liver. Still, some cancer cells express enzymes that efficiently metabolise fructose as an alternative fuel source. In addition, excessive fructose uptake has been linked to intestinal tumorigenesis as fructose is converted to fatty acids required for cancer cell growth [5]. Of note, it was proposed that high rates of *fructolysis* can also lead to ATP depletion due to the fast phosphorylation of fructose itself via fructokinase, and this, in turn, enhances the rate of glycolysis, which is instead inhibited when ATP levels are high. Intriguingly, fructose 1-phosphate can inhibit the glycolytic enzyme pyruvate kinase M2, promoting hypoxic cell survival in the intestine [6]. While fructose metabolism has been investigated in colorectal cancer, less is known about its role in other cancer types. In their manuscript, both Wang and Zhao provide an elegant characterisation of fructose metabolism in pancreatic and lung cancer, respectively, revealing new facets of this pathway.

In the study by Wang et al. [2], they first observed the upregulation of the fructose transporter GLUT5 and AKR1B1 in pancreatic cancer tissues in comparison to their normal counterparts. In addition, a panel of pancreatic cancer cells increased their proliferation rate when subject to increasing levels of fructose. They also observed that fructose is metabolised via *fructolysis* in pancreatic cancer cells expressing high levels of GLUT5, such as the MIA-Paca2, but not in those that exhibit low levels of this transporter, such as HPAC cells. Intriguingly, the latter have increased levels of expression of AKR1B1 and, consistently, can convert glucose into fructose via the *polyol pathway*. These results indicate that pancreatic cancer cells have engaged in distinct mechanisms to increase fructose metabolism: some express the transporter to use circulating fructose, while others can generate fructose via the polyol pathway. In both cases, inhibiting or silencing the respective fructose-generating strategies led to decreased proliferation in vivo. The authors then investigated possible connections between fructose metabolism and malignancy. They made the intriguing observation that fructose metabolism promotes tumour angiogenesis, as underlined by the expression of CD31 and VEGF in tumours from tumour-bearing mice exposed to fructose in the drinking water. Of note, this effect appeared mediated by small molecules secreted in the media of cancer cells exposed to fructose, and, consistently, the inhibition of fructose metabolism reduces vasculature formation in vitro. While the molecular determinants of the connection between fructose metabolism and angiogenesis remain to be fully demonstrated, the authors have shown that both lactate and amino acids could be responsible for the pro-angiogenic effects of fructose. Considering that these are end products of both fructose and glucose, it will be interesting to determine whether their kinetics, relative distribution, or other specific intermediates explain these new facets of fructose metabolism.

In the study by Zhao et al. [3], the researchers focused on the polyol pathway and, specifically, AKR1B1, which they found upregulated in a panel of human tumours and cancer cell lines, along with other enzymes associated with this pathway. Of relevance, they found that lung cancer cell lines A549 have the highest rate of endogenous production of fructose. They then performed some elegant metabolic tracing experiments by manipulating the levels of AKR1B1. They confirmed that this enzyme indeed allows the conversion of fructose-derived carbons into lactate, proving support to the notion that the *polyol pathway* is intact and bypasses some negative feedback checkpoints that occur in glycolysis. The silencing of AKR1B1 led to growth inhibition in vivo, indicating that the metabolic alterations the *polyol pathway* sustains are required for tumour cell growth and survival. Zhao et al. provide some mechanistic insights into how the inhibition of fructose metabolism causes tumour growth suppression. First, they show that the deletion of AKR1B1 causes cell cycle arrest and apoptosis. Second, they demonstrate that the inhibition of the *polyol pathway* causes the suppression of the RhoA-ROCK2 axis, which is involved in cell migration and metastasis. How fructose or some of its intermediary catabolites regulate cell cycle and cell migration has not been investigated in this paper. However, previous observations suggest that fructose metabolism could activate mTORC1 [7, 8], which is known to regulate cell cycle [9] and RhoA signalling [10]. It would be interesting to determine to what extent mTORC1 activity regulates these functional outcomes of fructose metabolism and the molecular mechanisms of sensing.

The growing research on fructose metabolism in cancer reveals a previously overlooked aspect of tumour biology and metabolism. Fructose, whether obtained from the diet or produced endogenously via the *polyol pathway*, provides cancer cells with a flexible energy source, particularly in stressful conditions where other nutrients and oxygen are limited. Besides energy, there is compelling evidence of an intriguing connection between fructose and other hallmarks of cancer, including angiogenesis, cell cycle progression, and cell migration (Fig. 1B). As our understanding of fructose’s role in cancer progresses, new therapeutic opportunities may arise, offering novel ways to disrupt cancer metabolism and improve patient outcomes.
